# The Swedish Version of the Multidimensional Inventory for Religious/Spiritual Well-Being: First Results From Swedish Students

**DOI:** 10.3389/fpsyg.2021.783761

**Published:** 2021-11-09

**Authors:** Magdalena Wenzl, Jürgen Fuchshuber, Nikita Podolin-Danner, Giorgia Silani, Human-Friedrich Unterrainer

**Affiliations:** ^1^Faculty of Psychology, University of Vienna, Vienna, Austria; ^2^CIAR: Center for Integrative Addiction Research, Grüner Kreis Society, Vienna, Austria; ^3^Department of Philosophy, University of Vienna, Vienna, Austria; ^4^Department of Psychiatry and Psychotherapeutic Medicine, Medical University of Graz, Graz, Austria; ^5^Department of Religious Studies, University of Vienna, Vienna, Austria

**Keywords:** religiosity, spirituality, personality factors, sense of coherence, scale development

## Abstract

**Background:** Studies investigating the relationship between religiosity/spirituality and mental health have suggested both positive and negative associations, highlighting the importance of multifaceted assessment of these rather broad constructs. The present study aims at contributing to this field of research by providing a validated Swedish version of the Multidimensional Inventory for Religious/Spiritual Well-Being (MI-RSWB-S) and further examining how this instrument relates to Big Five personality factors, Sense of Coherence (SOC), and religiosity.

**Methods:** Data were collected from a total of 1,011 Swedish students (747 females; age range 18–40) *via* completion of an online survey, including a new Swedish Version of the MI-RSWB-S, the Ten Item Personality Inventory (TIPI), the Sense of Coherence Scale (SOC-13), and the Centrality of Religiosity Scale (CRS-5).

**Results:** Results revealed adequate estimates of internal consistency and substantial evidence for the postulated six-dimensional structure. However, confirmatory factor analysis yielded poor fit indices, resulting in the development and validation of a revised measure of Religious/Spiritual Well-Being (RSWB), comprising the subscales General Religiosity and Connectedness. Most of the MI-RSWB-S dimensions were positively correlated with the personality domains Extraversion, Openness to Experience, Conscientiousness, and Agreeableness and negatively related to Neuroticism. SOC was positively linked to Hope Immanent, Forgiveness, Hope Transcendent, and Experiences of Sense of Meaning, whereas CRS exhibited positive correlations with all MI-RSWB-S subscales except Hope Transcendent.

**Conclusion:** The findings of the current study support the validity and reliability of the Swedish adoption of the MI-RSWB and confirm previously reported associations with the Big Five personality traits, SOC, and CRS. More in general, our results underline the putative substantial link between RSWB dimensions and mental health. Further research especially in clinical surroundings as well as by employing more representative samples is now warranted.

## Introduction

Sweden has been described as one of the most secular countries in the world (Esmer and Pettersson, 2007), with low levels of church attendance and belief in God, heaven and hell, the existence of sin, salvation, life after death, and the divinity of the Bible (Zuckerman, 2020). In a study comparing the importance of religion, worship attendance, frequency of prayer, and belief in God in 34 European countries, Sweden was ranked among the bottom 10 countries in all categories. Only 10% of the Swedish respondents stated that religion is very important in their lives, and about the same percentage of subjects claimed to pray daily (11%) and attend worship services at least monthly (11%). About 14% expressed that they believe in God with absolute certainty. Using an overall combined index, Sweden came in 30th place, with 10% of the sample defined as highly religious (Pew Research Center, 2018a). Consistent with global observations, Swedish men and young adults tend to be less religious than their female and older counterparts (Pew Research Center, 2016, 2018b). Despite low levels of religiosity and religious participation, accompanied by a continuous decline in membership, the Church of Sweden has approximately 5.7 million members, accounting for more than half of the Swedish population (Kyrkan, 2021). Moreover, although Swedish people generally hold sceptical and critical attitudes towards religion, relatively few call themselves convinced atheists. Many believe in “something” and possess an individualistic, non-dogmatic, and spiritual outlook on life (Thurfjell, 2019). The majority of the ones who do believe in God refer to a vague, distant entity rather than to biblical conceptions of God as an absolute, vengeful, or merciful being (Zuckerman, 2020).

Studies examining associations between religiosity/spirituality (R/S) and psychological health have yielded mixed results, linking R/S to various parameters of increased (e.g., high levels of well-being and meaning and purpose) as well as decreased mental health or mood pathology (e.g., cognitive rigidity, excessive concern over sins, and delayed professional psychological treatment; Koenig et al., 2012). The relationship between R/S and mental health is substantially dependent on the operationalisation of these rather “fuzzy” constructs (Zinnbauer et al., 1997; see also Hodapp and Zwingmann, 2019). Hence, its thorough investigation calls for the utilisation of instruments designed to capture the complexity of R/S contents (Koenig, 2008).

The Multidimensional Inventory for Religious/Spiritual Well-Being (MI-RSWB) is a multidimensional instrument for the assessment of Religious/Spiritual Well-Being (RSWB), defined as “the ability to experience and integrate meaning and purpose in existence through a connectedness with self, others, or a power greater than oneself” (Unterrainer et al., 2011, p. 117). The MI-RSWB comprises 48 items equally divided into six subscales: General Religiosity (GR), Connectedness (CO), Hope Transcendent (HT), Hope Immanent (HI), Forgiveness (FO), and Experiences of Sense and Meaning (SM). The dimension GR relates to traditional religious beliefs and institutionalised religion. CO refers to spiritual inclination and the feeling of being immersed in something bigger than oneself. The subscale HT reflects one’s hope for a better afterlife, whereas HI describes the extent to which one is hopeful for a better future here on earth. FO refers to the ability to extend forgiveness to oneself and others and to resign oneself to things that have gone wrong. The dimension SM pertains to meaningful life experiences, including those of honesty, gratitude, and true friendship. The first three subscales (GR, CO, and HT) can be used as parameters for the transcendent area of well-being [sub-score Transcendent Well-Being (TWB)], while the remaining subscales (HI, FO, and SM) relate to the immanent area of perception [sub-score Immanent Well-Being (IWB)]. In addition, the six dimensions can be summarised into a total score, thus providing a global measure of RSWB (Unterrainer et al., 2012).

The development of the MI-RSWB was initiated as a response to R/S needs of clinical and non-clinical populations and involved theoretical considerations in terms of an integration of a R/S dimension in the biopsychosocial model of health and illness (Engel, 1977). Furthermore, the MI-RSWB may be thought of as a multidimensional alternative to the two-dimensional Spiritual Well-Being Scale (SWBS), originated by Ellison (1983) (see also Moberg, 1971). The subscales of the SWBS, Religious Well-Being (RWB), and Existential Well-Being (EWB), are reflected in the differentiation of TWB and IWB. However, the inclusion of six dimensions allows for a more detailed examination of these areas of perception. The very content of these subscales was a result of interdisciplinary group discussion, literature research, and statistical analysis of empirical data (Unterrainer, 2021).

The original Austrian–German version of the inventory has been applied in various clinical and non-clinical studies. The latter resulted in norm values for the Austrian general population, with appealing psychometric properties for the subscales and the total score (Unterrainer and Fink, 2013). So far, the questionnaire has been translated into and validated in English (Unterrainer et al., 2012), Italian (Stefa-Missagli et al., 2014), Mexican Spanish (Berger et al., 2016), Bosnian (Malinovic et al., 2016), Russian (Agarkov et al., 2018), and Farsi (Dadfar et al., 2019), thereby demonstrating satisfactory psychometric properties (with one exception for HT in Dadfar et al., 2019). However, confirmatory factor analysis of data obtained in Austrian (Unterrainer et al., 2010) and British samples (Unterrainer et al., 2012) revealed only limited empirical support for the original MI-RSWB structure.

The MI-RSWB has been related to a number of psychosocial measures, including the “Big Five”-Factor Model (FFM), as most prominently described by Costa and McCrae (1992a). Studies investigating its associations with these personality domains have found positive correlations between extraversion and RSWB, HI, and SM (e.g., Berger et al., 2016) and negative links between Neuroticism and RSWB, whereas GR, CO, and SM proved to be unrelated to this specific trait (e.g., Malinovic et al., 2016). The personality dimension Openness to Experience has been consistently positively linked with SM and, in some cases, with the MI-RSWB total score, GR, HI, and CO (e.g., Hiebler-Ragger et al., 2018). Moreover, positive relationships have been noted between all MI-RSWB measures and both conscientiousness and agreeableness (e.g., Unterrainer et al., 2012). Similar results have been reported in other studies examining the relationship between R/S and these two personality traits (see Saroglou, 2010). What is more, substantial positive correlations have been observed between sense of coherence (SOC) and all MI-RSWB dimensions but CO, with the strongest association found for HI (e.g., Unterrainer et al., 2010). Finally, the MI-RSWB has been proven to be significantly related to other prominent measures of religiosity, such as the “Centrality of Religiosity” C-Scale (CRS; Huber and Huber, 2012). Thereby, for instance, Berger et al. (2016) reported positive links between CRS and all MI-RSWB scores except HT.

The current study aims are two-fold: As a first step, it is intended to introduce an internally validated Swedish version of the MI-RSWB-S. As a second step (external validation), it is planned to relate the MI-RSWB-S to established measures of the Big Five personality traits, SOC, and religiosity.

## Materials and Methods

### Participants and Procedure

This study is based on a convenience sample of Swedish students. The inclusion criteria for the study were: (1) Swedish citizenship, (2) fluency in the Swedish language, (3) student enrolment at a Swedish university or university college, and (4) being between 18 and 40years of age. Furthermore, a minimum survey completion time criterion of 4min was implemented, leading to the exclusion of one respondent.

Data were acquired between March 8 and April 5, 2021, by means of an online survey, using the web-based software tool SoSci Survey. Participants were primarily recruited through Facebook groups (> 230 groups) and Instagram accounts connected to Swedish universities and university colleges. These were instructed to download a PDF file containing comprehensive information about the study and indicated their consent to participate in it by checking a “yes” box before gaining access to the survey. Subjects were not compensated for their involvement in the research project. Ethical approval for the study was granted by the Ethics Committee of the University of Vienna.

### Psychometric Assessment

#### Multidimensional Inventory for Religious/Spiritual Well-Being

The original Austrian–German version of the MI-RSWB (Unterrainer et al., 2010) was translated into Swedish by a native Swedish-speaking psychology student fluent in German (M.W.). A back-translation was provided by a Swedish–German bilingual speaker, showing a high level of equivalence with the original instrument. The Swedish adoption of the MI-RSWB (MI-RSWB-S) comprises 48 items rated using a six-point Likert scale ranging from 1 (totally disagree) to 6 (totally agree). The items are equally distributed among the six subscales GR (e.g., “My faith gives me a feeling of security.”), FO (e.g., “There are things which I cannot forgive,” with reverse coding), HI (e.g., “I view the future with optimism.”), CO (e.g., “I have experienced the feeling of being absorbed into something greater.”), HT (e.g., “I often think about the fact that I will have to leave behind my loved ones.,” with reverse coding), and Experiences of SM (e.g., “I have experienced true (authentic) feelings.”). The total list of items of the MI-RSWB-S is presented in Supplementary Material.

High internal consistency has been reported for the original scale (*α*=0.89 for the total RSWB score and *α*≥0.73 for the subscales; Unterrainer et al., 2010) as well as for the English (Unterrainer et al., 2012), Italian (Stefa-Missagli et al., 2014), Mexican Spanish (Berger et al., 2016), Bosnian (Malinovic et al., 2016), and Russian adoptions (Agarkov et al., 2018), revealing Cronbach’s *α* coefficients of at least 0.83 with respect to the total score.

#### Ten Item Personality Inventory

The Ten Item Personality Inventory (TIPI) is a brief measure of the Big Five personality domains of the Five-Factor Model (FFM; Costa and McCrae, 1992a), namely Extraversion, Neuroticism, Openness to Experience, Conscientiousness, and Agreeableness. Extraversion includes traits related to sociability, activity, and positive affectivity, while Neuroticism describes the individual tendency to experience psychological distress. Openness to Experience refers to the extent to which an individual is intellectually curious, behaviourally flexible, emotionally differentiated, and non-dogmatic as well as sensitive to imagination, art, and beauty. Conscientiousness reflects the degree to which a person is scrupulous, well-organized, and diligent. Individuals who score high on Agreeableness are trusting, sympathetic, and cooperative, whereas people with a low level of Agreeableness tend to be cynical, callous, and antagonistic.

The TIPI assesses Extraversion, Emotional Stability (reversed Neuroticism), Openness to Experience, Conscientiousness, and Agreeableness with two items per personality dimension (e.g., “I see myself as extraverted, enthusiastic” for Extraversion). All items are responded to on a Likert scale ranging from 1 (disagree strongly) to 7 (agree strongly). Owing to the small number of items, some of the TIPI subscales have demonstrated low internal consistency. The highest Cronbach’s *α* coefficient has been obtained for Emotional Stability (*α*=0.73), followed by Extraversion (*α*=0.68), Conscientiousness (*α*=0.50), Openness to Experience (*α*=0.45), and Agreeableness (*α*=0.40; Gosling et al., 2003). However, the TIPI has reached satisfactory levels of convergent and discriminant validity when related to the Big-Five Inventory (BFI; John and Srivastava, 1999), thereby displaying convergences (*r*=0.65–0.87; *p*<0.01; mean *r*=0.77) comparable to those of the well-established multi-item instruments Trait Descriptive Adjectives (TDA; Goldberg, 1992; mean *r*=0.81) and NEO Five-Factor Inventory (NEO-FFI; Costa and McCrae, 1992b; mean *r*=0.73), and discriminant correlations below 0.37 (absolute mean *r*=0.20). Furthermore, the subscales have shown adequate test–retest reliability (*r*=0.62–0.77; mean *r*=0.72; Gosling et al., 2003). The Swedish version of the TIPI has been provided by Lundell (2014).

#### Sense of Coherence Scale

The Sense of Coherence 13-item scale (SOC-13) is a short version of the original Sense of Coherence Scale (SOC-29) used to measure levels of Sense of Coherence (SOC), the core concept of Antonovsky’s salutogenic model (Antonovsky, 1987). SOC pertains to a global orientation which expresses an individual’s ability to cope with stress and to stay healthy (Mittelmark et al., 2017). It comprises three components: Comprehensibility, Manageability, and Meaningfulness. Comprehensibility refers to the perception that internal and external stimuli are structured, predictable, and explicable, while Manageability reflects the perceived availability of resources to deal with the demands presented by the stimuli. Meaningfulness describes the extent to which these demands are seen as challenges worthy of personal commitment. In a broader sense, Meaningfulness relates to the feeling that life makes sense and has emotional meaning (Antonovsky, 1987, 1991b).

The SOC-13 utilises a seven-point Likert scale with varying verbal response anchors to capture Comprehensibility (five items), Manageability (four items), and Meaningfulness (four items; e.g., “Do you have the feeling that you really do not care about what is going on around you?”). The internal consistency of the SOC-13 scale has been investigated in an exhaustive amount of studies, with Cronbach’s *α* values ranging from 0.70 to 0.92 (Eriksson and Lindström, 2005). Swedish studies have reported good internal consistency for the general population (e.g., Larsson and Kallenberg, 1999) as well as for adolescents and young adults (Räty et al., 2003). The Swedish translation of the SOC-13 (KASAM-13) has been published in Antonovsky (1991a).

#### Centrality of Religiosity Scale

The Centrality of Religiosity Scale (CRS) is an instrument for assessing the centrality, importance, or salience of religious meanings in personality (Huber and Huber, 2012). It captures five core dimensions of religiosity, namely Public Practice, Private Practice, Religious Experience, Ideology, and Intellect. Public Practice reflects the extent to which one integrates their religious life in a social organism by for instance participating in public religious rituals or activities, whereas Private Practice relates to activities and rituals of personal devotion to a transcendent sphere of reality (e.g., prayer and meditation). The dimension of religious experience includes individual experiences and feelings of being connected to an ultimate reality. Ideology refers to religious beliefs, convictions, and patterns of plausibility (e.g., with respect to the existence of God), while Intellect pertains to religious knowledge and interest, hermeneutical skills, and ways of thinking.

The Swedish version of the CRS-5 (CRS-5 SWE) has been provided by Sjöborg (2014). It measures the five dimensions of religiosity with one item each. These items are rated on a five-point (1–5; Ideology, Intellect, and Religious Experience), six-point (1–6; Public Practice), or eight-point Likert scale (1–8; Private Practice; “How often do you pray?”) with different verbal response anchors, together with a “do not know” option. Data collected by means of six-point and eight-point response formats are recoded into values between 1 and 5. The composite score of the CRS-5 has demonstrated high internal consistency (*α*=0.85; Huber and Huber, 2012).

### Statistical Analysis

Data were analysed in three stages. First, a principal component analysis (PCA) with VARIMAX rotation was conducted using the first 300 responses (exploration phase sample). As a second step, confirmatory factor analyses (CFA) were carried out on a different sample, as suggested by Boateng et al. (2018), comprising the remaining data set (*n*=711; validation phase sample). Model fit was considered acceptable if the following criteria were met: (a) Comparative Fit Index (CFA)>0.90, (b) Tucker–Lewis Index (TLI)>0.90, (c) Normed Fit Index (NFI)>0.90, and (d) Square Root Error Approximation (RMSEA)<0.08 with the upper bound of the 90% CI<0.10 (Kline, 2016). Third, descriptive statistics of the total sample (*N*=1,011) were generated to provide an overview of the MI-RSWB-S scores. In addition, independent *t* tests and Pearson’s correlations were performed to examine gender and age effects and how the MI-RSWB-S measures relate to each other and the validation scales (TIPI, SOC-13, and CRS-5). Cronbach’s *α* coefficients were calculated to determine the internal consistency of these instruments, following the guidelines provided by George and Mallery (2016). PCA, descriptive statistics, *t* tests, reliability analysis, and Pearson’s correlations were conducted *via* SPSS 25, whereas the CFAs were computed using AMOS 26.

## Results

### Sample Characteristics

A total of 1,011 participants were included in the final study sample. The sociodemographic characteristics of the total sample (*N*=1,011), exploration phase sample (*n*=300), and the validation phase sample (*n*=711) are given in Table 1. Almost three-quarters (73.9%) of the subjects in the total sample identified as female, and more than three-quarters (77.9%) were between the ages of 20 and 29 (*M*=24.78; *SD*=4.83). Nine hundred and two respondents (89.2%) were born in Sweden. All 21 counties were represented in the sample, with a relatively high proportion of subjects coming from Stockholm (21.6%) and Västra Götaland (20.6%). Data were obtained from students from a total of 36 university/university colleges (e.g., the University of Gothenburg, Uppsala University, and Stockholm University). Most of the participants were single (45.9%), living together with their partner (22.9%) or in a relationship (18.2%). Less than 10 percent (9.5%) had biological children (*M*=0.19; *SD*=0.65). Almost half of the respondents (46.2%) were members of the Church of Sweden, and approximately one-sixth (16.6%) belonged to another Christian denomination. About the same number of subjects had never been part of (16.4%) or left (15.4%) a religious community. Less than 10 percent (7.0%) labelled themselves as Muslims, predominately as adherents to Sunni Islam. Relatively few belonged to a Jewish (1.3%), Buddhist (1.3%), or Hindu (0.2%) community. The participants using the category “others” (1.0%) described their religious affiliation as follows: Druze, Forn Sed, Luciferian, Satanic Temple, Mandaeism, Sikhism, Yogi, and Wicca.

**Table 1:** Sample characteristics.

Variable	Total sample (*N*=1,011) *n* (%)	Exploration phase sample (*n*=300) *n* (%)	Validation phase sample (*n*=711) *n* (%)
**Gender**			
Female	747 (73.9)	217 (72.3)	530 (74.5)
Male	252 (24.9)	79 (26.3)	173 (24.3)
Other	12 (1.2)	4 (1.3)	8 (1.1)
**Age**			
18–19	63 (6.2)	22 (7.3)	41 (5.8)
20–24	525 (51.9)	142 (47.3)	383 (53.9)
25–29	263 (26.0)	81 (27.0)	182 (25.6)
30–34	100 (9.9)	36 (12.0)	64 (9.0)
35–40	60 (5.9)	19 (6.3)	41 (5.8)
**Place of birth**			
East Sweden (SE1)	355 (35.1)	93 (31.0)	262 (36.8)
South Sweden (SE2)	391 (38.7)	116 (38.7)	275 (38.7)
North Sweden (SE3)	156 (15.4)	66 (22.0)	90 (12.7)
Other Nordic country	11 (1.1)	2 (0.7)	9 (1.3)
Other European country (outside the Nordics)	35 (3.5)	7 (2.3)	28 (3.9)
Other country (outside Europe)	63 (6.2)	16 (5.3)	47 (6.6)
**Relationship status**
Married	91 (9.0)	26 (8.7)	65 (9.1)
Engaged	45 (4.5)	13 (4.3)	32 (4.5)
Cohabitation	232 (22.9)	74 (24.7)	158 (22.2)
In a relationship	184 (18.2)	59 (19.7)	125 (17.6)
Single	464 (45.9)	128 (42.7)	336 (47.3)
Divorced	8 (0.8)	4 (1.3)	4 (0.6)
**Religious affiliation**			
Christianity (Church of Sweden)	467 (46.2)	145 (48.3)	322 (45.3)
Christianity (free church)	102 (10.0)	36 (12.0)	66 (9.3)
Christianity (Eastern Orthodox)	33 (3.3)	5 (1.7)	28 (3.9)
Christianity (Roman Catholic)	30 (3.0)	5 (1.7)	25 (3.5)
Christianity (other)	3 (0.3)	0 (0.0)	3 (0.4)
Islam (Sunni)	59 (5.8)	10 (3.3)	49 (6.9)
Islam (Shia)	10 (1.0)	2 (0.7)	8 (1.1)
Islam (other)	2 (0.2)	1 (0.3)	1 (0.1)
Judaism	13 (1.3)	6 (2.0)	7 (1.0)
Buddhism	13 (1.3)	3 (1.0)	10 (1.4)
Hinduism	2 (0.2)	0 (0.0)	2 (0.3)
Never been a part of a religious community	166 (16.4)	55 (18.3)	111 (15.6)
Left a religious community	156 (15.4)	50 (16.7)	106 (14.9)
Other	10 (1.0)	1 (0.3)	9 (1.3)

SE1, SE2, and SE3 refer to the first-level nomenclature of territorial units for statistics (NUTS) regions of Sweden.

### Principal Component Analysis

A PCA with orthogonal rotation (VARIMAX with Kaiser normalisation) was conducted on the 48 items of the MI-RSWB-S in the exploration phase sample (*n*=300). Based on theoretical considerations regarding the dimensional structure of the MI-RSWB, the number of extracted components was set to 6. Sampling adequacy was evaluated through assessment of the Kaiser-Meyer-Olkin (KMO) index and Bartlett’s test of sphericity. The KMO measure was 0.89, well above the recommended threshold of 0.60 (Kaiser, 1974), and all KMO values for individual items were greater than 0.62, thus exceeding the acceptable limit of 0.50 (Kaiser and Rice, 1974). Furthermore, Bartlett’s test of sphericity was significant (*χ*^2^_(1128)_=8065.11, *p*<0.001), indicating suitability of the data for PCA. As given in Table 2, the six-component solution accounted for 53.14% of the total variance. GR explained the largest proportion of the variance.

**Table 2:** Six-component solution for the Swedish version of the MI-RSWB (MI-RSWB-S).

Principal component	Component loadings		Eigenvalue	% of Variance	Cumulative %
	Range	*M*			
GR	0.80–0.91	0.86	8.91	18.56	18.56
HI	0.44–0.79	0.64	4.45	9.27	27.83
FO	0.47–0.80	0.65	4.31	8.98	36.81
HT	0.18–0.72	0.52	2.78	5.78	42.59
SM	0.22–0.67	0.40	2.72	5.66	48.25
CO	0.03–0.64	0.40	2.35	4.89	53.14

Principal component analysis with VARIMAX rotation with Kaiser normalisation (*n*=300). GR, General Religiosity; HI, Hope Immanent; FO, Forgiveness; HT, Hope Transcendent; SM, Experiences of Sense and Meaning; and CO, Connectedness.

### Confirmatory Factor Analysis

**Figure 1:**
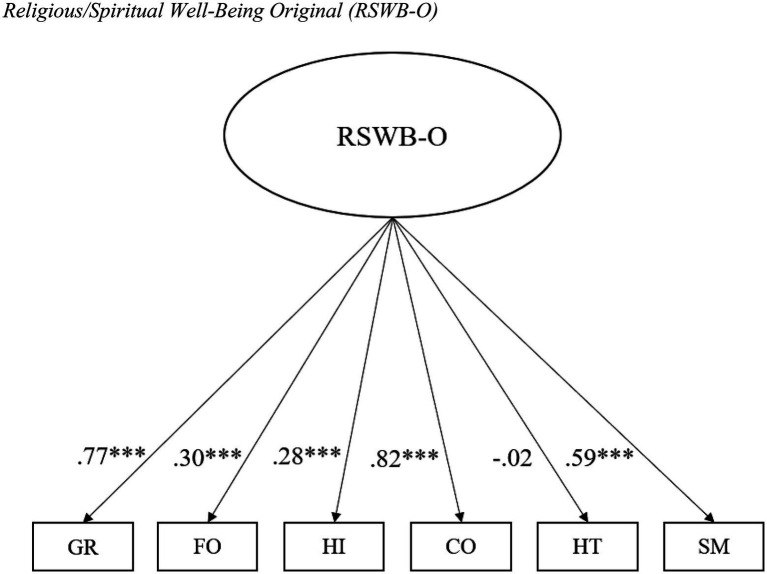
Religious/Spiritual Well-Being (RSWB) Original (RSWB-O). Confirmatory factor analysis (*n*=711). GR, General Religiosity; FO, Forgiveness; HI, Hope Immanent; CO, Connectedness; HT, Hope Transcendent; SM, Experiences of Sense and Meaning; and RSWB-O, Religious/Spiritual Well-Being Original. ^***^p<0.001.

As shown in Figure 1, a CFA was performed on the validation phase sample (*n*=711) to test the factorial structure of the MI-RSWB-S. The CFA yielded incremental fit indices below the acceptable level of 0.90 (*CFA*=0.82; *TLI*=0.70; *NFI*=0.81) and a RMSEA value exceeding 0.08 with a 90% CI upper limit greater than 0.10 (*RMSEA*=0.15; 90% CI=0.13–0.16), indicating poor fit. Moreover, RSWB [hereinafter referred to as RSWB Original (RSWB-O)] was unrelated to HT (*β*=−0.02) and weakly linked to HI (*β*=0.28) and FO (*β*=0.30; *p*<0.001 for all values). These results provide little support for the proposed model, prompting further analysis.

**Figure 2:**
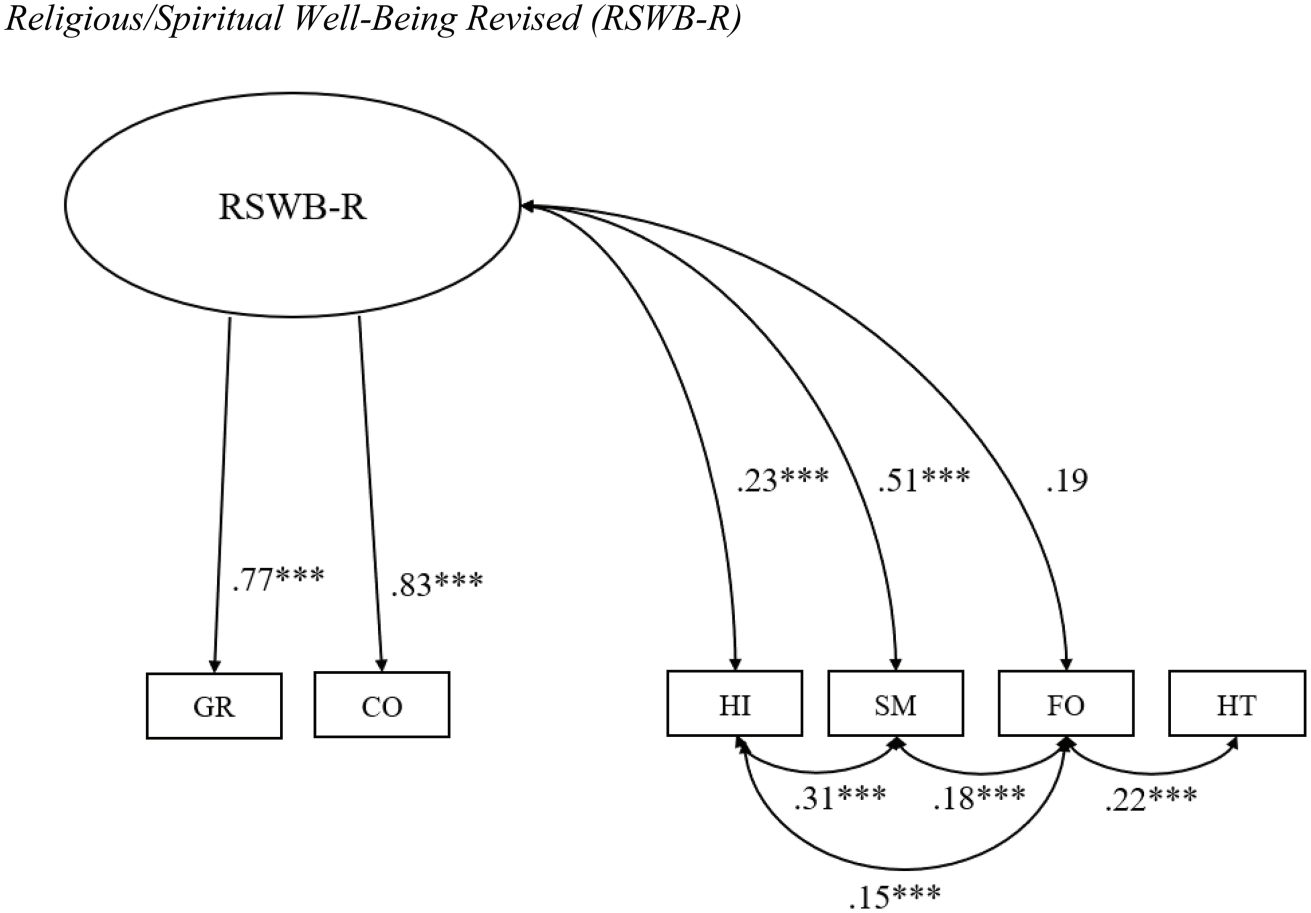
Religious/Spiritual Well-Being Revised (RSWB-R). Confirmatory factor analysis (*n*=711). GR, General Religiosity; CO, Connectedness; HI, Hope Immanent; SM, Experiences of Sense and Meaning; FO, Forgiveness; HT, Hope Transcendent; and RSWB-R, Religious/Spiritual Well-Being Revised. ^***^p<0.001.

Based on conceptual considerations and empirical findings, we developed a revised model (see Figure 2) in which GR and CO are summarised into RSWB [RSWB Revised (RSWB-R)], while HI, SM, FO, and HT operate as independent factors. The CFA for the revised model resulted in incremental fit indices above 0.90 (*CFI*=0.995; *TLI*=0.980; *NFI*=0.990) and a RMSEA below 0.08, with a 90% CI upper bound smaller than 0.10 (*RMSEA*=0.039; 90% CI=0.000–0.077). In addition, the Chi-square test was non-significant (*χ2*=8.42; *p*>0.05; *χ2/df*=17.71). Furthermore, RSWB-R displayed strong associations with GR (*β*=0.77) and CO (*β*=0.83; *p*<0.001 for all calculations). Taken together, these findings lend support for the postulated structure.

### Descriptive Statistics

Table 3 presents descriptive statistics for the MI-RSWB-S in the total sample (*N*=1,011). FO, HI, HT, and SM were negatively skewed, representing a predominance of high scores, while GR, CO, RSWB-O, and RSWB-R displayed positive skewness, reflecting negative response patterns. Platykurtic distribution was observed for all measures but HI. Normal distribution was assessed by inspecting the absolute values of skewness and kurtosis. All values fell within the acceptable range of ±2 (George and Mallery, 2016).

**Table 3:** Descriptive statistics of the Swedish version of the MI-RSWB (MI-RSWB-S).

	Female (*n*=747)		Male (*n*=252)		Total (*N*=1,011)		min	max	*z*_skewness_	*z*_kurtosis_
	*M*	*SD*	*M*	*SD*	*M*	*SD*
GR	21.20	14.45	20.09	13.45	20.96	14.19	8	48	9.16	−7.27
FO	34.58	8.48	34.14	8.80	34.50	8.53	10	48	−6.06	−3.49
HI	35.06	6.53	33.54	6.96	34.62	6.66	9	48	−8.19	3.39
CO	22.44	8.96	21.85	9.00	22.36	8.99	8	48	7.79	−2.33
HT	33.65	7.57	35.44	7.38	34.15	7.55	10	48	−5.04	−1.83
SM	36.07	6.19	36.13	6.40	36.08	6.25	15	48	−4.08	−1.77
RSWB-O	182.99	32.66	181.19	32.31	182.67	32.50	98	269	4.77	−3.79
RSWB-R	43.64	21.29	41.94	20.93	43.32	21.21	16	93	7.31	−6.79

*N*=1,011. min, minimum score; max, maximum score; GR, General Religiosity; FO, Forgiveness; HI, Hope Immanent; CO, Connectedness; HT, Hope Transcendent; SM, Experiences of Sense and Meaning; RSWB-O, Religious/Spiritual Well-Being Original; and RSWB-R, Religious/Spiritual Well-Being Revised.

### Gender and Age Effects

Furthermore, independent *t* tests were conducted to assess differences between women (*n*=747) and men (*n*=252). Women demonstrated significantly higher scores (*M*=35.06; *SD*=6.53) than men (*M*=33.54; *SD*=6.96) on HI [*t*(997)=3.14; *p*<0.01], representing an effect size of *d*=0.22. Moreover, women scored lower (*M*=33.65; *SD*=7.57) than men (*M*=35.44; *SD*=7.38) on HT [*t*(997)=−3.26; *p*<0.01], with an effect size of *d*=0.24. No other gender effects were observed.

In addition, Pearson’s correlations were calculated to examine age effects, revealing positive associations between age and CO (*r*=0.15; *p*<0.01), SM (*r*=0.13; *p*<0.01), and RSWB-O (*r*=0.08; *p*<0.05).

### Intercorrelations and Internal Consistencies

As given in Table 4, the MI-RSWB-S demonstrated acceptable to excellent internal consistency for all measures except SM, with Cronbach’s *α* coefficients ranging from 0.67 to 0.97. RSWB-O was significantly positively correlated with RSWB-R and all six MI-RSWB-S dimensions, displaying the strongest associations with RSWB-R, GR, and CO. RSWB-R was positively related to all subscales but HT, with the strongest link found for GR. Positive relationships were established between all subscales, with the exception of HT, which was only linked to FO. The strongest subscale intercorrelation was observed between GR and CO, followed by SM and CO (*p*<0.01).

**Table 4:** Intercorrelations and internal consistencies of the MI-RSWB-S.

Dimension	α	GR	FO	HI	CO	HT	SM	RSWB-O	RSWB-R
GR	0.97	-	0.36^**^	0.20^**^	0.66^**^	−0.00	0.41^**^	0.83^**^	0.95^**^
FO	0.85		-	0.13^**^	0.17^**^	0.25^**^	0.18^**^	0.59^**^	0.32^**^
HI	0.81			-	0.21^**^	0.05	0.33^**^	0.46^**^	0.23^**^
CO	0.81				-	−0.06	0.51^**^	0.74^**^	0.86^**^
HT	0.77					-	−0.04	0.28^**^	−0.03
SM	0.67						-	0.62^**^	0.49^**^
RSWB-O	0.90							-	0.87^**^
RSWB-R	0.94								-

*N*=1,011. GR, General Religiosity; FO, Forgiveness; HI, Hope Immanent; CO, Connectedness; HT, Hope Transcendent; SM, Experiences of Sense and Meaning; RSWB-O, Religious/Spiritual Well-Being Original; and RSWB-R, Religious/Spiritual Well-Being Revised.

** *p*<0.01.

### MI-RSWB-S in Relation to Personality, Sense of Coherence, and Religiosity

Internal consistencies of the TIPI subscales, SOC-13, and CRS-5 as well as associations between these validation instruments and the MI-RSWB-S are given in Table 5.

**Table 5:** MI-RSWB-S in relation to personality traits, Sense of Coherence, and religiosity.

	*α*	GR	FO	HI	CO	HT	SM	RSWB-O	RSWB-R
Extraversion	0.75	0.04	0.09^**^	0.24^**^	0.11^**^	0.09^**^	0.18^**^	0.18^**^	0.08^*^
Neuroticism	0.70	−0.07^*^	−0.17^**^	−0.30^**^	−0.03	−0.22^**^	−0.11^**^	−0.22^**^	−0.06
Openness	0.40	0.11^**^	0.14^**^	0.08^*^	0.24^**^	0.11^**^	0.24^**^	0.24^**^	0.18^**^
Conscientiousn.	0.57	0.08^*^	0.13^**^	0.33^**^	0.02	0.06	0.10^**^	0.18^**^	0.06
Agreeableness	0.24	0.13^**^	0.29^**^	0.21^**^	0.10^**^	0.09^**^	0.19^**^	0.26^**^	0.13^**^
SOC	0.83	0.06	0.31^**^	0.48^**^	0.00	0.30^**^	0.14^**^	0.30^**^	0.04
CRS	0.92^a^	0.94^**^ ^b^	0.36^**^ ^b^	0.17^**^ ^b^	0.64^**^ ^b^	−0.01^b^	0.39^**^ ^b^	0.79^**^ ^b^	0.90^**^ ^b^

*N*=1,011. GR, General Religiosity; FO, Forgiveness; HI, Hope Immanent; CO, Connectedness; HT, Hope Transcendent; SM, Experiences of Sense and Meaning; RSWB-O, Religious/Spiritual Well-Being Original; RSWB-R, Religious/Spiritual Well-Being Revised; Neuroticism, reversed Emotional Stability; Openness, Openness to Experience; Conscientiousn, Conscientiousness; SOC, Sense of Coherence; and CRS, Centrality of Religiosity Scale.

a *n*=948.

b *n*=1004.

* *p*<0.05;

** *p*<0.01.

The TIPI subscales Extraversion (*α*=0.75) and Neuroticism (*α*=0.70) displayed acceptable levels of internal consistency, whereas Conscientiousness (*α*=0.57) showed poor internal consistency. The lowest Cronbach’s *α* values were found for Agreeableness (*α*=0.24) and Openness to Experience (*α*=0.40).

Extraversion was positively correlated with RSWB-O (*p*<0.01), RSWB-R (*p*<0.05), and all MI-RSWB-S dimensions (*p*<0.01) but GR, to which it was unrelated. Neuroticism was negatively linked to GR (*p*<0.05), FO, HI, HT, SM, and RSWB-O (*p*<0.01) and unassociated with CO and RSWB-R, whereas Openness to Experience and Agreeableness were positively related to all MI-RSWB-S measures (*p*<0.01; *p*<0.05 for Openness to Experience and HI). Conscientiousness exhibited positive correlations with GR (*p*<0.05), FO, HI, SM, and RSWB-O (*p*<0.01). No significant associations were found between Conscientiousness and the other MI-RSWB-S scores. SOC-13 demonstrated good internal consistency, with a Cronbach’s *α* coefficient of 0.83. SOC was positively correlated with FO, HI, HT, SM, and RSWB-O (*p*<0.01) and unrelated to GR, CO, and RSWB-R. Excellent internal consistency was obtained for CRS-5 (*α*=0.92). Positive correlations were observed between CRS and all MI-RSWB-S measures (*p*<0.01) except HT.

## Discussion

The main objective of the present work was to provide a validated Swedish version of the MI-RSWB-S. Furthermore, it was intended to investigate how the MI-RSWB-S relates to the Big Five personality traits, SOC, and CRS. Using data from 1,011 Swedish students, a psychometric evaluation of the translated instrument was undertaken. Thereby, we observed acceptable to excellent internal consistency for most of the subscales, with the highest Cronbach’s *α* coefficient found for GR, which mirrors the results of previous research (e.g., Unterrainer et al., 2010, 2012). While the postulated six-component solution of the MI-RSWB received considerable empirical support based on PCA results, CFA of the original factor structure demonstrated poor model fit. On the basis of these findings, together with those of earlier reports and theoretical considerations regarding the conceptualisation of RSWB, a new model was specified. CFA of the suggested structure yielded excellent model fit indices, thereby confirming its construct validity.

In light of these results, we propose a revision of the MI-RSWB structure. Instead of summarising all MI-RSWB dimensions into a total score and using this as an estimate of RSWB (RSWB-O), RSWB may now be obtained by computing the subscales GR and CO (RSWB-R). Subsequently, GR can be calculated for the assessment of RWB, while CO may be used as a measure of Spiritual Well-Being (SWB). Nevertheless, HI, SM, FO, and HT can be analysed independently to gain insight into these specific facets of well-being.

Descriptive analysis of the collected data revealed notable differences in response patterns on the MI-RSWB-S dimensions with a predominance of low GR and CO scores and a preponderance of high values on the other subscales. These findings are markedly different from those obtained among students from other countries (e.g., Unterrainer et al., 2010, 2012; Malinovic et al., 2016), which have demonstrated higher levels of homogeneity within the subscales as well as higher GR and CO mean scores. However, at least, the predominance of low GR values in the current sample is coherent with the notion of Sweden as a relatively secular country (Esmer and Pettersson, 2007) with a small proportion of highly religious people (Pew Research Center, 2018a). Furthermore, our findings underline the importance of multidimensional assessment of R/S by suggesting that low levels of GR and CO (and consequently RSWB-R) do not rule out the possibility of extending forgiveness and experiencing hope and sense and meaning. This raises the question of what motivational factors other than those of explicitly R/S kind may be responsible for this observation. For example, a person practicing the virtue of forgiveness may not attribute this behaviour to religious beliefs. Although significant, a meta-analysis (Fehr et al., 2010) investigating the correlates of forgiveness reported a relatively weak positive link between forgiveness and religiosity, especially when compared to dispositional and situational factors such as state empathy and apology. Moreover, an atheist may score high on HT as a result of accepting the mortal nature of human existence, rather than as an expression of confidence in life after death. Similarly, the optimistic expectations and sense of certainty about the future captured by the dimension HI may not stem from religious convictions or spiritual experiences (see e.g., Benzein et al., 2000). In fact, researchers have identified a number of predictors of hope, including life satisfaction, optimism, self-esteem, and social support (Yarcheski and Mahon, 2016).

In line with past research (e.g., Malinovic et al., 2016; Hiebler-Ragger et al., 2018), Extraversion was positively related to HI, SM, and RSWB-O, with the highest correlation found for HI. Contrary to most other observations (e.g., Berger et al., 2016), a significant positive link was identified between Extraversion and HT. Largely consistent with previous reports (e.g., Agarkov et al., 2018; Hiebler-Ragger et al., 2018), HI, HT, and RSWB-O were identified as the strongest negative correlates of Neuroticism. Also in accord with these studies as well as with those investigating the relationship between Neuroticism and spirituality (see Saroglou, 2010), no significant association was found between this personality trait and CO. However, Neuroticism was significantly negatively related to GR and SM, which has not been noted in other studies (e.g., Unterrainer et al., 2012). Openness to Experience was positively linked to all MI-RSWB-S measures. These findings are somewhat different from those of preceding studies, which have generated mixed results. Substantially in agreement with earlier observations (e.g., Malinovic et al., 2016), Conscientiousness exhibited significant positive correlations with GR, FO, HI, SM, and RSWB-O and was unrelated to CO and HT. The strongest link was found between Conscientiousness and HI, partly consistent with previous results (e.g., Stefa-Missagli et al., 2014). In accordance with the findings of Unterrainer et al. (2012), Agreeableness was positively associated with all MI-RSWB-S scores, with the highest correlations observed between Agreeableness and FO (consistent with the findings of Fehr et al., 2010), RSWB-O, and HI. Whereas RSWB-O was significantly related to all of the Big Five traits (thereby exhibiting positive correlations to all measures but Neuroticism), RSWB-R was only (positively) associated with Extraversion, Openness to Experience, and Agreeableness. Moreover, these correlations were weaker than those found for RSWB-O. However, it is important to note that only Extraversion and Neuroticism demonstrated acceptable levels of internal consistency. Consequently, only the reported links between these personality traits and the MI-RSWB-S measures can be interpreted with a relatively high degree of certainty. Conscientiousness, on the other hand, showed poor internal consistency, albeit slightly higher than that reported in Gosling et al. (2003). The Cronbach’s *α* values obtained for Openness to Experience and Agreeableness were lower than those previously observed and indicated unacceptable levels of internal consistency. Nevertheless, the correlations identified between the MI-RSWB-S scores and Conscientiousness, Openness to Experience, and Agreeableness were somewhat similar to those found in other studies.

Sense of Coherence was positively correlated with all MI-RSWB-S measures but GR, CO, and RSWB-R. These results indicate that FO, HI, CO, HT, and SM may be more connected to SOC than R/S in a narrower sense, thus drawing attention to specific aspects of R/S rather than to its conceptual core. The highest correlation was observed between SOC and HI. These findings are substantially in line with those of preceding studies (e.g., Unterrainer et al., 2010; Berger et al., 2016), which have reported positive links between SOC and RSWB-O and all its facets except CO.

Centrality of Religiosity Scale was positively related to all MI-RSWB-S subscales but HT, to which it was unrelated. Mirroring the results reported by Berger et al. (2016), CRS was most strongly associated with GR, followed by CO, SM, FO, and HI. This supports the notion that GR represents a more general measure of religiosity. Furthermore, CRS was more strongly related to RSWB-R than to RSWB-O.

Moreover, small gender effects were identified for immanent and transcendent hope, with women scoring higher than men on HI and lower on HT, as observed by Unterrainer and Fink (2013). Unlike previous studies, which have reported higher levels of RSWB-O, FO (e.g., Unterrainer and Fink, 2013; Stefa-Missagli et al., 2014), GR (e.g., Berger et al., 2016), CO, and SM (e.g., Unterrainer and Fink, 2013) in women than in men (see also Pew Research Center, 2016); no further gender differences were found. In addition, weak correlations were detected between age and CO, SM, and RSWB-O.

## Limitations and Future Perspectives

Despite the strengths of the present work (e.g., large sample size, multidimensional assessment of R/S, and exhaustive statistical analysis), several limitations warrant mention. First, our sample comprises Swedish students with a high proportion of female respondents and a significant number of participants under the age of 30. Given these circumstances, this sample cannot be regarded as representative for the Swedish population. Second, the survey was distributed on Facebook and Instagram, consequently excluding students who are not active on these platforms. It should also be noted that this research was conducted during the Covid-19 pandemic, potentially further limiting the generalisability of the findings. Third, in view of the cross-sectional nature of the study, no causal inferences can be drawn. Last, although some of the TIPI subscales demonstrated remarkably low levels of internal consistency, no alternative estimates of reliability (e.g., test-retest reliability correlations) were provided, as suggested by Gosling et al. (2003). To circumvent some of these limitations, future research might use longer personality measures and focus on more representative samples and longitudinal analyses. Studies may also be conducted in clinical populations. In addition to this, qualitative research is encouraged to explore the mechanisms responsible for the observed discrepancies between the subscale scores of the MI-RSWB-S. Considering the positive links between SOC and HI, CO, HT, and SM, future studies may also delve into the reasons and implications of these observations. Moreover, further research might consider examining how the MI-RSWB-S relates to other measures associated with mental health, such as for instance the components of the PERMA model of well-being (Seligman, 2011).

In conclusion, the Swedish adoption of the MI-RSWB demonstrated psychometric properties equivalent to those of the original Austrian–German version. However, CFA favoured a two-factor model over the original six-dimensional structure, resulting in a revision of the inventory. This revised version of the MI-RSWB-S can be regarded as a valid and reliable instrument for assessing RSWB and consequently a valuable contribution to the field of psychology of religion, which may be used to further investigate the relationship between R/S and mental health.

## Data Availability Statement

The raw data supporting the conclusions of this article will be made available by the authors, without undue reservation.

## Ethics Statement

The studies involving human participants were reviewed and approved by University of Vienna. The patients/participants provided their written informed consent to participate in this study.

## Author Contributions

MW and H-FU conceptualised the study. MW and JF acquired the data and performed all statistical analyses. All authors critically discussed the results. The manuscript was drafted by MW and revised by H-FU, JF, and NP-D. GS proof read the manuscript. All authors contributed to the article and approved the submitted version.

## Conflict of Interest

The reviewer HB-C declared a shared affiliation, with one of the authors H-FU to the handling editor at the time of the review.

The remaining authors declare that the research was conducted in the absence of any commercial or financial relationships that could be construed as a potential conflict of interest.

## Publisher’s Note

All claims expressed in this article are solely those of the authors and do not necessarily represent those of their affiliated organizations, or those of the publisher, the editors and the reviewers. Any product that may be evaluated in this article, or claim that may be made by its manufacturer, is not guaranteed or endorsed by the publisher.

## References

[ref1] Agarkov V. A. Alexandrov Y. I. Bronfman S. A. Chernenko A. M. Kapfhammer H.-P. Unterrainer H.-F. (2018). A Russian adaptation of the multidimensional inventory for religious/spiritual well-being: psychometric properties for Young adults and associations with personality and psychiatric symptoms. Arch. Psychol. Relig. 40, 104–115. doi: 10.1163/15736121-12341347

[ref2] Antonovsky A. (1987). Unraveling the Mystery of Health: How People Manage Stress and Stay Well. San Francisco, CA: Jossey-Bass.

[ref3] Antonovsky A. (1991a). Hälsans mysterium. Stockholm: Natur och kultur.

[ref4] Antonovsky A. (1991b). “The structural sources of salutogenic strengths,” in Personality and Stress: Individual Differences in the Stress Process. eds. Cooper C. L. Payne R. (Chichester: John Wiley & Sons), 67–104.

[ref5] Benzein E. G. Saveman B.-I. Norberg A. (2000). The meaning of Hope in healthy, nonreligious swedes. West. J. Nurs. Res. 22, 303–319. doi: 10.1177/01939450022044430, PMID: 10804894

[ref6] Berger D. Fink A. Perez Gomez M. M. Lewis A. Unterrainer H.-F. (2016). The validation of a Spanish version of the multidimensional inventory of religious/spiritual well-being in Mexican college students. Span. J. Psychol. 19:E3. doi: 10.1017/sjp.2016.9, PMID: 26887859

[ref7] Boateng G. O. Neilands T. B. Frongillo E. A. Melgar-Quiñonez H. R. Young S. L. (2018). Best practices for developing and validating scales for health, social, and Behavioral research: A primer. Front. Public Health 6:149. doi: 10.3389/fpubh.2018.00149, PMID: 29942800 PMC6004510

[ref8] Costa P. T. McCrae R. R. (1992a). Normal personality assessment in clinical practice: The NEO personality inventory. Psychol. Assess. 4, 5–13. doi: 10.1037/1040-3590.4.1.5

[ref9] Costa P. T. McCrae R. R. (1992b). Revised NEO Personality Inventory (NEO-PI-R) and NEO Five-Factor Inventory (NEO-FFI): Professional Manual. Odessa, FL: Psychological Assessment Resources, Inc.

[ref10] Dadfar M. Lester D. Turan Y. Beshai J. A. Unterrainer H.-F. (2019). Validation of the multidimensional inventory for religious spiritual well-being with Iranian samples. Ment. Health Relig. Cult. 22, 591–601. doi: 10.1080/13674676.2019.1628194

[ref11] Ellison C. W. (1983). Spiritual well-being: conceptualization and measurement. J. Psychol. Theol. 11, 330–338. doi: 10.1177/009164718301100406

[ref12] Engel G. L. (1977). The need for a new medical model: A challenge for biomedicine. Science 196, 129–136. doi: 10.1126/science.847460, PMID: 847460

[ref13] Eriksson M. Lindström B. (2005). Validity of Antonovsky’s sense of coherence scale: A systematic review. J. Epidemiol. Community Health 59, 460–466. doi: 10.1136/jech.2003.018085, PMID: 15911640 PMC1757043

[ref14] Esmer Y. Pettersson T. (eds.) (2007). Measuring and Mapping Cultures: 25 Years of Comparative Value Surveys. Leiden: Brill.

[ref15] Fehr R. Gelfand M. J. Nag M. (2010). The road to forgiveness: A meta-analytic synthesis of its situational and dispositional correlates. Psychol. Bull. 136, 894–914. doi: 10.1037/a0019993, PMID: 20804242

[ref16] George D. Mallery P. (2016). IBM SPSS Statistics 23 Step by Step: A Simple Guide and Reference, 14th Edn. New York, NY: Routledge.

[ref17] Goldberg L. R. (1992). The development of markers for the big-five factor structure. Psychol. Assess. 4, 26–42. doi: 10.1037/1040-3590.4.1.26

[ref18] Gosling S. D. Rentfrow P. J. Swann W. B. (2003). A very brief measure of the big-five personality domains. J. Res. Pers. 37, 504–528. doi: 10.1016/S0092-6566(03)00046-1

[ref19] Hiebler-Ragger M. Fuchshuber J. Dröscher H. Vajda C. Fink A. Unterrainer H. -F. (2018). Personality influences the relationship between primary emotions and religious/spiritual well-being. Front. Psychol. 9:370. doi: 10.3389/fpsyg.2018.00370, PMID: 29615950 PMC5868126

[ref800] Hodapp B. Zwingmann C. (2019). Religiosity/spirituality and mental health: A meta-analysis of studies from the German-speaking area. J. Relig. Health. 58, 1970–1998. doi: 10.1007/s10943-019-00781-2 30632002

[ref20] Huber S. Huber O. W. (2012). The centrality of religiosity scale (CRS). Religion 3, 710–724. doi: 10.3390/rel3030710

[ref21] John O. P. Srivastava S. (1999). “The big five trait taxonomy: history, measurement, and theoretical perspectives,” in Handbook of Personality: Theory and Research. eds. Pervin L. A. John O. P. (New York, NY: Guilford Press), 102–138.

[ref22] Kaiser H. F. (1974). An index of factorial simplicity. Psychometrika 39, 31–36. doi: 10.1007/BF02291575

[ref23] Kaiser H. F. Rice J. (1974). Little jiffy, mark IV. Educ. Psychol. Meas. 34, 111–117. doi: 10.1177/001316447403400115

[ref24] Kline R. B. (2016). Principles and Practice of Structural Equation Modeling, 4th Edn. New York, NY: Guilford Press.

[ref25] Koenig H. G. (2008). Concerns about measuring “spirituality” in research. J. Nerv. Ment. Dis. 196, 349–355. doi: 10.1097/NMD.0b013e31816ff796, PMID: 18477877

[ref26] Koenig H. G. King D. E. Carson V. B. (2012). Handbook of Religion and Health, 2nd Edn. New York, NY: Oxford University Press.

[ref27] Kyrkan S. (2021). Svenska kyrkan i siffror. Available at: https://www.svenskakyrkan.se/statistik (Accessed September 5, 2021).

[ref28] Larsson G. Kallenberg K. (1999). Dimensional analysis of sense of coherence using structural equation modelling. Eur. J. Personal. 13, 51–61. doi: 10.1002/(SICI)1099-0984(199901/02)13:1<51::AID-PER321>3.0.CO;2-P

[ref29] Lundell E. (2014). Ten-Item Personality Inventory-(TIPI) – Swedish translation. Available at: http://gosling.psy.utexas.edu/wp-content/uploads/2014/09/TIPISwedishtranslation.doc (Accessed September 5, 2021).

[ref30] Malinovic A. Fink A. Lewis A. J. Unterrainer H. -F. (2016). Dimensions of religious/spiritual well-being in relation to personality and stress coping: initial results from bosnian young adults. J. Spiritual. Ment. Health 18, 43–54. doi: 10.1080/19349637.2015.1059301

[ref31] Mittelmark M. B. Sagy S. Eriksson M. Bauer G. F. Pelikan J. M. Lindström B.. (eds.) (2017). The Handbook of Salutogenesis. Cham: Springer. 28590610

[ref32] Moberg D. O. (1971). Spiritual Well-Being: Background and Issues. White House Conference on Aging. Washington, DC: U.S. Government Printing Office.

[ref33] Pew Research Center. (2016). The Gender Gap in Religion Around the World. Available at: https://www.pewforum.org/wp-content/uploads/sites/7/2016/03/Religion-and-Gender-Full-Report.pdf (Accessed September 5, 2021).

[ref34] Pew Research Center. (2018a). Eastern and Western Europeans Differ on Importance of Religion, Views of Minorities, and Key Social Issues. Available at: https://www.pewforum.org/wp-content/uploads/sites/7/2018/10/Eastern-Western-Europe-FOR-WEB.pdf (Accessed September 5, 2021).

[ref35] Pew Research Center. (2018b). The Age Gap in Religion Around the World. Available at: https://www.pewforum.org/wp-content/uploads/sites/7/2018/06/ReligiousCommitment-FULL-WEB.pdf (Accessed September 5, 2021).

[ref36] Räty L. K. A. Larsson B. M. W. Söderfeldt B. A. (2003). Health-related quality of life in youth: A comparison between adolescents and young adults with uncomplicated epilepsy and healthy controls. J. Adolesc. Health 33, 252–258. doi: 10.1016/S1054-139X(03)00101-0, PMID: 14519566

[ref37] Saroglou V. (2010). Religiousness as a cultural adaptation of basic traits: a five-factor model perspective. Personal. Soc. Psychol. Rev. 14, 108–125. doi: 10.1177/1088868309352322, PMID: 20023209

[ref38] Seligman M. E. P. (2011). Flourish: A Visionary New Understanding of Happiness and Well-Being. New York, NY: Free Press.

[ref39] Sjöborg A. (2014). “CRS-5 SWE,” in Secular and Sacred?: The Scandinavian Case of Religion in Human Rights, Law and Public Space. (eds.) Breemer R. van den Casanova J. Wyller T. (Göttingen: Vandenhoeck & Ruprecht), 236–260.

[ref40] Stefa-Missagli S. Huber H. P. Fink A. Sarlo M. Unterrainer H. -F. (2014). Dimensions of religious/spiritual well-being, personality, and mental health: initial results from italian college students. Arch. Psychol. Relig. 36, 368–385. doi: 10.1163/15736121-12341290

[ref41] Thurfjell D. (2019). Det gudlösa folket: De postkristna svenskarna och religionen. Stockholm: Nordstedts.

[ref42] Unterrainer H. -F. (2021). The multidimensional measurement of religious/ spiritual well-being: recent developments in scale validation and clinical applications. submitted.

[ref43] Unterrainer H. -F. Fink A. (2013). Das Multidimensionale Inventar zum religiös-spirituellen Befinden (MI-RSB): Normwerte für die österreichische Allgemeinbevölkerung. Diagnostica 59, 33–44. doi: 10.1026/0012-1924/a000077

[ref44] Unterrainer H. -F. Huber H. -P. Ladenhauf K. H. Wallner-Liebmann S. J. Liebmann P. M. (2010). MI-RSB 48: Die Entwicklung eines multidimensionalen Inventars zum religiös-spirituellen Befinden. Diagnostica 56, 82–93. doi: 10.1026/0012-1924/a000001

[ref45] Unterrainer H. -F. Ladenhauf K. H. Wallner-Liebmann S. J. Fink A. (2011). Different types of religious/spiritual well-being in relation to personality and subjective well-being. Int. J. Psychol. Relig. 21, 115–126. doi: 10.1080/10508619.2011.557003

[ref46] Unterrainer H. -F. Nelson O. Collicutt J. Fink A. (2012). The English version of the multidimensional inventory for religious/spiritual well-being (MI-RSWB-E): first results from british college students. Religion 3, 588–599. doi: 10.3390/rel3030588

[ref47] Yarcheski A. Mahon N. E. (2016). Meta-analyses of predictors of hope in adolescents. West. J. Nurs. Res. 38, 345–368. doi: 10.1177/0193945914559545, PMID: 25421543

[ref48] Zinnbauer B. J. Pargament K. I. Cole B. Rye M. S. Butter E. M. Belavich T. G.. (1997). Religion and spirituality: Unfuzzying the fuzzy. J. Sci. Study Relig. 36, 549–564. doi: 10.2307/1387689

[ref49] Zuckerman P. (2020). Society without God. What the Least Religious Nations Can Tell us about Contentment, 2nd edn. New York, NY: New York University Press.

