# Supplementary material for: The Swedish Version of the Multidimensional Inventory for Religious/Spiritual Well-Being: First Results From Swedish Students
Source: Front Psychol. 2021 Nov 9;12:783761. doi: 10.3389/fpsyg.2021.783761 (PMC8630782; doi:10.3389/fpsyg.2021.783761)
Supplement: Supplementary file 1 [file Data_Sheet_1.docx]

# Appendix

**The Swedish version of the Multidimensional Inventory for Religious/Spiritual Well-Being (MI-RSWB-S)**

**Instructions [instruktioner]**

*Innan du besvarar frågorna ber jag dig lägga märke till följande: En stor del av frågorna kommer att röra din religiösa tro. Du kommer även att stöta på begreppet ”Gud”, när du går igenom de olika frågorna.*

*Här är det viktigt att påpeka:*

- Dina svar kommer endast att användas för vetenskapliga forskningsändamål och utvärderas **helt anonymt**.
- Denna undersökning är inte sponsrad av någon religiös förening eller gemenskap och inga uppgifter kommer heller att lämnas ut till en sådan.
- Begreppet ”Gud” **kan, men måste inte**, förstås enligt traditionell kristen trosuppfattning.
- Om du anser att begreppet ”Gud” är opassande, kan du tankemässigt ersätta det med en formulering som exempelvis ”högre makt”.
- Frågorna i detta formulär är möjliga att besvara även om du helt och hållet tar avstånd från religiös tro. En sådan hållning ges utrymme för i frågeformuläret.

*Vänligen besvara frågorna spontant och sitt inte för länge med en fråga.*

*Dessutom ber jag dig att besvara varje fråga, eftersom det bara då är möjligt att göra en korrekt utvärdering.*

| **List of items** | | |
| --- | --- | --- |
| Nr. Dim. Item | | |
| 1 | GR | Min tro ger mig en känsla av trygghet. |
| 7 | GR | Det är möjligt för mig att finna tillfredsställelse i förtroliga samtal med Gud. |
| 13 | GR | Jag kommer att kunna bemästra alla problem med Guds hjälp. |
| 19 | GR | I vissa stunder i mitt liv känner jag mig väldigt nära Gud. |
| 25 | GR | Med Guds hjälp kommer jag att bli lycklig igen. |
| 31 | GR | Jag vet att Gud är barmhärtig. |
| 37 | GR | Jag deltar gärna i religiösa sammankomster. |
| 43 | GR | Jag känner Guds närvaro i naturen. |
| 2 | FO | Det finns saker som jag inte kan förlåta.* |
| 8 | FO | Det finns människor som jag hatar.* |
| 14 | FO | Det finns människor som jag aldrig kommer att kunna förlåta.* |
| 20 | FO | Det finns saker som man inte ska förlåta.* |
| 26 | FO | När någon har sårat mig, försöker jag i regel hämnas.* |
| 32 | FO | Tanken på att se mina fiender lida tillfredsställer mig.* |
| 38 | FO | Det finns människor som förtjänar att bli illa behandlade.* |
| 44 | FO | Jag har förlåtit de som har sårat mig. |
| 3 | HI | Jag ser optimistiskt på framtiden. |
| 9 | HI | Jag tror att det kommer att bli bättre i framtiden. |
| 15 | HI | Jag tror att mitt liv rör sig i rätt riktning. |
| 21 | HI | Jag tror att jag i framtiden kommer att ha fler positiva än negativa upplevelser. |
| 27 | HI | Jag tror att jag i framtiden kommer leva så som jag föreställer mig det. |
| 33 | HI | Jag har en exakt bild av hur min framtid ska se ut. |
| 39 | HI | Min framtid tycks mig väldigt oviss.* |
| 45 | HI | Jag tror att framtiden har spännande utmaningar i beredskap för mig. |
| 4 | CO | Jag har varit med om att jag som person tycktes gå upp i något större. |
| 10 | CO | Jag tror att jag kommer att återfödas efter min död. |
| 16 | CO | Det finns människor som jag känner en övernaturlig koppling till. |
| 22 | CO | Jag har varit med om saker som har gjort mig medveten om att ingenting någonsin dör. |
| 28 | CO | Jag tror på en fortsatt existens efter döden. |
| 34 | CO | Jag har upplevt saker som inte kan uttryckas i ord. |
| 40 | CO | Jag har upplevt att det finns föremål som utstrålar en alldeles speciell kraft. |
| 46 | CO | Jag tror att jag i framtiden kommer att skaffa mig erfarenheter som många människor inte har möjlighet till. |
| 5 | HT | Jag tänker ofta på att jag kommer att behöva lämna efter mig mina nära och kära.* |
| 11 | HT | Jag skulle göra vad som helst för att förlänga livet för mina nära och kära.* |
| 17 | HT | Det är svårt för mig att tänka på att mina nära och kära en dag inte kommer att leva längre.* |
| 23 | HT | Jag är rädd för att bli bortglömd efter min död.* |
| 29 | HT | Jag skulle göra vad som helst för att förlänga mitt liv.* |
| 35 | HT | Jag är rädd för vad som kommer att hända med mig efter min död.* |
| 41 | HT | Med döden upphör allt hopp.* |
| 47 | HT | Jag är rädd för att få stå till svars för mina felsteg efter min död.* |
| 6 | SM | Jag har erfarenhet av äkta känslor. |
| 12 | SM | Jag har upplevt djup tillgivenhet. |
| 18 | SM | Jag har upplevt sann vänskap. |
| 24 | SM | Jag har ofta upplevt öppenhet och ärlighet. |
| 30 | SM | Jag har varit med om saker som jag vill uppleva om och om igen. |
| 36 | SM | Jag har ofta haft upplevelser som påverkat mig djupt. |
| 42 | SM | Jag har varit med om att jag kan uppslukas så mycket av något att jag glömmer allt runt omkring mig. |
| 48 | SM | Jag har haft en eller flera upplevelser som fått mig att inse meningen med livet. |
| *Note.* Nr. = item number; Dim. = dimension; GR = General Religiosity; FO = Forgiveness; HI = Hope Immanent; CO = Connectedness; HT = Hope Transcendent; SM = Experiences of Sense and Meaning. Likert scale: 1 totally disagree [stämmer inte alls] - 6 totally agree [stämmer helt och hållet].  * = coded inversely. | | |

| **Evaluation scheme** | |
| --- | --- |
| Dimension | Item number |
| General Religiosity (GR) [Allmän Religiositet (AR)] | 1, 7, 13, 19, 25, 31, 37, 43 |
| Forgiveness (FO) [Förlåtelse (FÖ)] | 2*, 8*, 14*, 20*, 26*, 32*, 38*, 44 |
| Hope Immanent (HI) [Hopp Immanent (HI)] | 3, 9, 15, 21, 27, 33, 39*, 45 |
| Connectedness (CO) [Samhörighet (SA)] | 4, 10, 16, 22, 28, 34, 40, 46 |
| Hope Transcendent (HT) [Hopp Transcendent (HT)] | 5*, 11*, 17*, 23*, 29*, 35*, 41*, 47* |
| Experiences of Sense and Meaning (SM) [Erfarenheter av Syfte och Mening (SM)] | 6, 12, 18, 24, 30, 36, 42, 48 |
| *Note.* Religious/Spiritual Well-Being [Religiöst/Andligt Välbefinnande (RAV)].  * = coded inversely. | |
